# 

**Published:** 1893-04

**Authors:** 


### CONTENTS

CLINICAL LECTURE—
Surgical Clinic at Rush Medical College. By John B. Hamilton, M. D., Chicago. 65
ORIGINAL COMMUNICATIONS—
Ascites in Connection with Gynecology. By Professor A. Gusserow, Berlin........ 69
Surgical Cases from Practice. By C. D. Hurt, M. D., Atlanta, Ga.................. 76
Report of a Case of Pyosalpinx Relieved by Drainage Through the Vagina. By Jeff S. Davis, M. D. Montevallo, Ala.......... 83
The Abuse of Astringents in the Treatment of Catarrh. By Charles M. Shields, M. D., Richmond, Va.......... 86
SELECTED ARTICLE—
Cocaine in Surgery. By Jules Anber, Paris.......... 88
SOCIETY REPORTS—
Richmond Academy of Medicine and Surgery.......... 93
CORRESPONDENCE—
Two Interesting Cases.......... 96
EDITORIAL—
The State Medical Association.......... 98
Opening of the Atlanta Polyclinic.......... 99
The Quack Doctor's Own Methods.......... 100
SELECTIONS AND ABSTRACTS.
Surgery.......... 103
Obstetrics and Gynecology.......... 107
General Medicine and Therapeutics.......... 113
Skin and Genito-Urinary Diseases.......... 117
Eye, Ear, Nose and Throat.......... 120
MEDICAL ITEMS.......... 122
BOOK REVIEWS.......... 124
INDEX TO ADVERTISEMENTS.......... xii
ITEMS OF INTEREST.......... x xi xxvi xxxi
PREMIUM OFFERS.......... xv

